# Prevalence, characteristics and treatment of concomitant injury to liver and spleen with vascular injury after blunt abdominal trauma

**DOI:** 10.1038/s41598-025-14113-w

**Published:** 2025-08-07

**Authors:** Anna Kistner, Zlatan Alagic, Anders Enocson, Seppo K. Koskinen

**Affiliations:** 1 Department of Molecular Medicine and Surgery, Karolinska Institute, Stockholm, 171 77 Sweden; 2 Department of Nuclear Medicine and Medical Physics, Karolinska University Hospital, Stockholm, 171 76 Sweden; 3 Department of Diagnostic Radiology, Karolinska University Hospital, Stockholm, 171 76 Sweden; 4 Department of Trauma, Acute surgery and Orthopaedics, Karolinska University Hospital, Stockholm, 171 76 Sweden; 5 Department of Clinical Science, Intervention, and Technology, Division for Radiology, Karolinska Institute, Stockholm, 171 77 Sweden

**Keywords:** Abdominal trauma, Liver-spleen injury, ISS, Active bleeding, Survival rate, Health care, Whole body imaging, Medical research, Outcomes research

## Abstract

Our purpose was to assess the prevalence of liver injuries as well as concomitant injuries to the liver and spleen in patients with blunt or penetrating abdominal trauma, and to determine the prevalence, management and outcome of active bleeding and contained vascular injuries (CVI; pseudoaneurysm/AV-fistula) seen on admission CT. A retrospective, single-center, longitudinal cohort study with nine-year data (2013–2021) of all ≥ 15-year-old patients with severe blunt or penetrating abdominal trauma and an ICD code for liver and/or splenic trauma. CT examinations were identified. Radiology, medical reports and images were reviewed and only patients with an adequate admission CT were included in the final study group. Of 2805 patients with abdominal trauma (71% males), 409 patients (14.6%) had a liver injury, and 329 had a CT on admission (329/409; 80.4%). 313 patients (11.2%) had a splenic injury and 262 had a CT (262/313; 83.7%). Of these, 65 patients or 2.3% (65/2805) had injury to both organs, with 49 patients with CT (49/65; 75.4%), combined group (CG) (79% males). The median (range) ISS was 21 (4–75) for single organ injury patients and 34 (9–75) for patients with both organs injured (*p* < 0.0001). Active liver or splenic bleeding was seen in 5.8% and 17.9%, respectively. In CG, 11 (11/49; 22.4%) patients had active bleeding. Of these, two patients had active bleeing in both organs (4.1%). Liver patients with active bleeding had significantly higher ISS (*p* = 0.025) than those without. In CG, ISS did not differ significantly between patients with and without active bleeding (*p* = 0.073), however, it tended to be higher in those with active bleeding. Most liver injuries with active bleeding were treated non-operatively (12/19; 63.2%). An active bleeding was more common in spleen than in liver patients; odds ratio (OR) (95% CI) 3.57 (2.04–6.25), *p* < 0.0001. A CVI was more common in splenic compared with liver injuries, OR 6.71 (95% CI; 2.27–19.9, *p* < 0.0001). Active bleeding was more common in CG than in single organ injury patients; OR 3.67 (1.73–7.79), *p* < 0.0016. 30-day survival rate did not differ between patients with or without active bleeding, but was slightly lower in CG compared with only liver injury (89.8% vs. 93.7%, *p* = 0.36). In conclusion the prevalence of liver injury in abdominal trauma seen on admission CT was 11.7% of all patients with blunt or penetrating abdominal trauma, and concomitant splenic and liver injury was seen in 1.7%. Non-operative management was applied in almost two thirds of patients with liver injuries. Active bleeding was seen in 5.8% of liver, 17.9% of splenic and 22.4% of CG patients. ISS was doubled in CG compared with single organ injury patients. Active bleeding was more common in CG, and CG had slightly increased mortality rate compared with single organ injury patients.

**Supplementary Information:**

The online version contains supplementary material available at 10.1038/s41598-025-14113-w.

## Introduction

Trauma is a common cause of death in Europe, especially in the younger population. In Sweden, 3.5% of all deaths in 2022 were trauma related^1^. Deaths due to falls represented most of these deaths in both men and women, and have increased over time for older men.

Abdominal trauma constitutes a certain entity of all traumas, with its true incidence estimated to be 6.2% in a large trauma cohort^2^. Injuries to the spleen and the liver are the most common organ injuries in major abdominal trauma with an incidence of approximately 50–60%^3^, the majority of which are caused by blunt trauma.

The imaging method of choice in major blunt trauma is currently CT with intravenous contrast^4^. In major abdominal organ trauma, the severity of both splenic and liver injuries are classified according to the AAST (American Association for the Surgery of Trauma) Injury Grading Scale. During the past decades, the management of liver injuries has shifted from exploratory laparotomy to a usually non-operative observational management^5^. In abdominal trauma patients, mortality rates have declined over time in a Northern European setting between 2004 and 2018, probably attributed to improvements in diagnostics and therapeutics^2^. Thus, the outcome during the last decades has improved, and non-operative management has often proved to be an effective treatment^6^. In liver injuries, most patients with grade 1 to 3 injuries are treated non-operatively, whereas higher grading often requires surgery^7^. Hemodynamics and associated injuries may determine if a patient is managed conservatively or surgically. Studies have shown that higher grade splenic injury is associated with failure in non-operative management, in contrast to higher grade liver injuries that did not show this association^8^. With the introduction of angioembolization, the need for open surgical intervention has further diminished. According to the the latest World Society of Emergency Surgery (WSES) liver trauma management algorithm, hemodynamically stable adult patients with a contrast blush should undergo angioembolization^9^. Similarly, in splenic trauma cases, the WSES algorithm recommends angioembolization for adult patients with AAST grade IV-V injuries and a contrast blush^10^.

The combined trauma to both liver and spleen occurs more frequently in severe trauma and treatment is highly resource demanding^11^. The incidence of concomitant injury to both liver and spleen in the acute phase is not well known, but it has been reported to occur in up to 9–11% of patients with either splenic or liver trauma^3,12^. In a cohort of splenic and/or liver injuries, the incidence of combined injuries was 12.6%^13^. It is crucial for the trauma leader and the radiologist to be able to outline factors that increase the risk of concomitant injury, facilitating their detection and the provision of necessary treatment. Moreover, in abdominal trauma, bleeding with severe blood loss can lead to death. Timely localization of the bleeding source can improve the efficacy of patient management^14^. As whole body CT has an established role in trauma imaging, radiologists may have a life-saving role in detecting active bleeding requiring immediate intervention. Therefore, knowledge of the prevalence of active bleeding may help the on-call radiologist interpret the images in critical situations.

The main purpose of this study was to investigate the prevalence of liver injuries and concomitant injuries to the liver and spleen in patients with blunt or penetrating abdominal trauma seen on admission CT at a single level 1 trauma center during a nine-year period (2013–2021). We aimed to compare the characteristics and outcomes between single organ injury (in liver or spleen) versus combined organ injury (in liver and spleen). Secondary purposes included studying the prevalence, management and outcome of active bleeding and/or contained vascular injury (CVI; pseudoaneurysm/AV-fistula) seen on admission CT.

## Material & methods

**Fig. 1:**
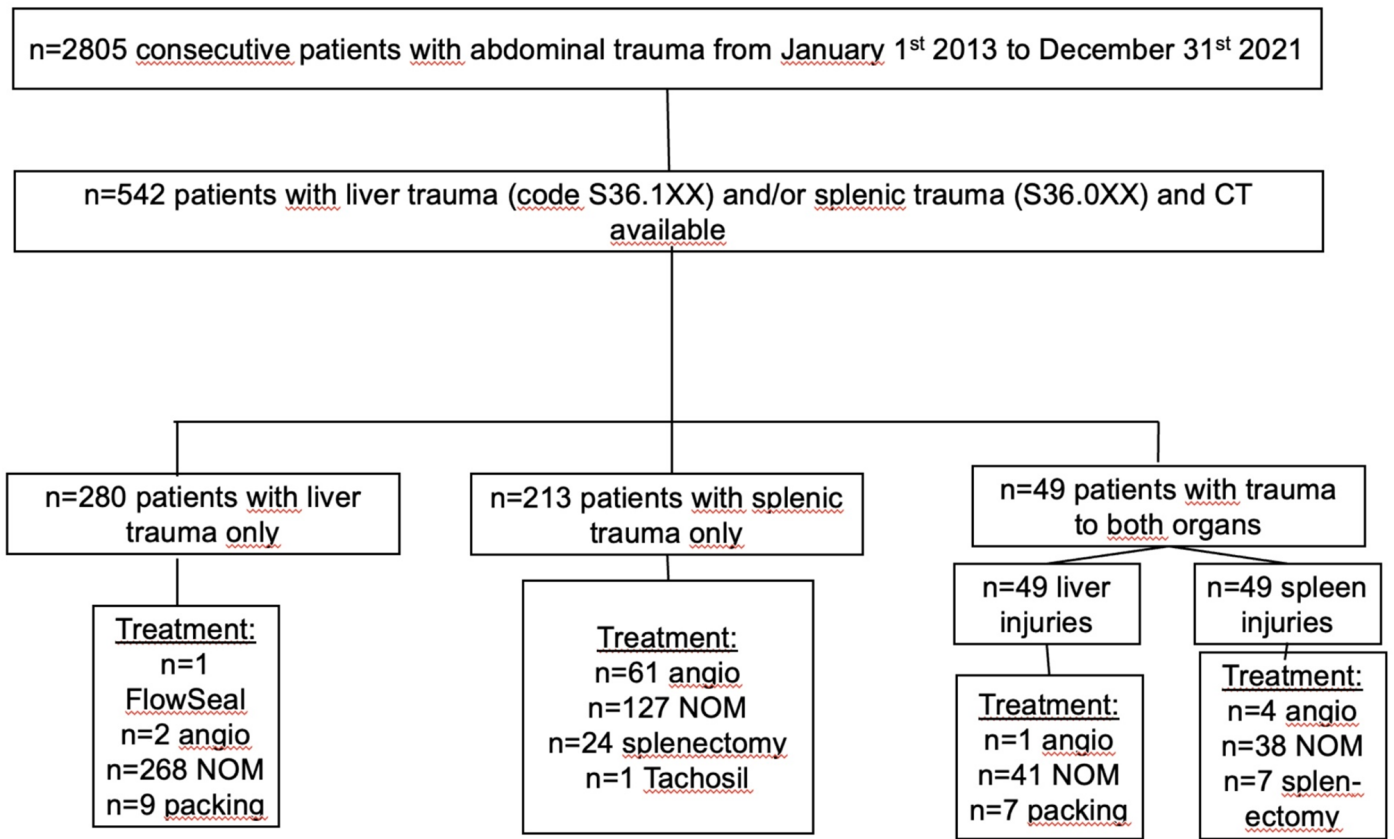
Liver and splenic injuries in patients with abdominal trauma at Karolinska University Hospital between 2013–2021.

Approval from the local ethics committee was obtained for this retrospective, longitudinal cohort study (Ethical approval number 2019–05643, 2022-02753-02) and due to its retrospective design, informed consent was waived. The collection of patients and data has been earlier described^15^. In brief, nine-year data (2013–2021) of all patients with severe blunt or penetrating abdominal trauma were retrieved from the local trauma register of a level 1 trauma center (Fig. 1). All patients, 15-years and older with an ICD code for liver (S36.1XX) and/or splenic trauma (S36.0XX) were included. The following parameters were extracted from the trauma register and/or electronic medical records: age, gender, Injury Severity Score (ISS), New Injury Severity Score (NISS), 30-day mortality, injury mechanism, surgical and/or radiological interventions (angioembolization), and length of hospital stay (LOS). Injury mechanism: fall from height, was divided into high height (high-energy), and fall in the same plane (low-energy). The CT images were retrieved from the local picture archiving and communication systems (the radiological information system (RIS)/picture archiving and communication system (PACS); SECTRA AB, Linköping, Sweden), and the CT protocol was recorded.

At our institution treatment decisions for blunt or penetrating liver injuries are made on a case-by-case basis considering a combination of clinical parameters and imaging findings. In general, stable patients without contrast extravasation are treated with non-operative management (NOM). For stable patients with a contrast blush surgical treatment is considered. Hemodynamically unstable patients receive interventional or surgical treatment.

CT and angiographic examinations from all eligible patients were identified and the reports and images were reviewed by a senior radiologist (SKK) with 30 years of experience. An eligible admission CT was a CT performed according to the trauma CT protocol, see a detailed description below. All CT scans had previously been interpreted by at least one radiology resident and a senior radiologist. The final study group comprised patients with an adequate admission CT available and a CT or surgically verified liver or spleen injury.

The liver injuries seen on CT were graded according to AAST Injury Grading Scale 1994 and 2018 revisions. A vascular injury was defined as either an active bleeding (contrast extravasation) or a CVI^12^. If a CVI was suspected, a second radiologist reviewed the cases, and any discrepancy was solved by consensus. The detailed description of splenic trauma has been reported earlier^13^.

The number of active bleedings as well as treatment based on electronic medical records and/or available angioembolization data in PACS was assessed.

### CT technique

At our hospital, the multitrauma imaging was conducted on a 256-slice multi-detector CT (MDCT) (Revolution CT, GE Healthcare, Milwaukee, Wisconsin, USA) and a 64-slice MDCT scanner (LightSpeed VCT, GE Healthcare, Milwaukee, Wisconsin, USA). During 2015 the Revolution CT scanner (RevCT) was installed and replaced the LightSpeed VCT scanner (VCT) in our trauma department. Concomitantly our single-phase (venous) standard trauma CT protocol was modified to a multi-phase protocol by including a whole-body CT in arterial phase for high-level trauma patients. For low-energy trauma patients, a single-phase (venous) trauma CT protocol was used on the RevCT. In the multi-phase trauma CT protocol on the RevCT, the software SmartPrep™ (GE Healthcare) was used to time the arterial phase and the delay of the venous phase was 45.0 s after the end of the arterial phase. In the single-phase trauma CT protocol on the RevCT, the delay was 65.0 s, and on the VCT 55.0–60.0 s. The iodinated contrast agents ioversol (350 mg/ml) or iohexol (350 mg/ml) were used with a flow rate of approximately 4.5-5 ml/s in the CT protocol that included an arterial phase and 2.5–3.5 ml/s in the venous phase only CT protocols.

### Statistics

The Mann-Whitney U test was used for the comparison of continuous data. The chi-square test was used to compare categorical data. Odds ratio was used to analyze the frequency of active bleeding or CVI in splenic injuries compared with liver injuries, the differences in the frequency of active bleeding between single organ and combined organ injuries, and survival rates in combined injuries compared with the single liver or spleen organ injury group. Statistical analyses were done using a commercial software package SAS/STAT v.9.4 (SAS Institute Inc., Cary, NC, USA).

## Results

### Patient and injury characteristics

Patient characteristics are summarized in Table 1. Of 2805 patients with abdominal trauma in the trauma register (29% females), 409 patients (409/2805; 14.6%) had a liver injury of whom 329 underwent CT (329/409; 80.4%) and 313 (313/2805; 11.2%) had a splenic injury; of these, 262 underwent CT (262/313; 83.7%). In total 591 patients had a liver and/or splenic injury. 592 patients (592/2805; 21.1%) had a single organ injury to either liver or spleen, and 493 of these underwent CT. 344 patients had an injury in liver only, with 280 undergoing CT, while 248 had an injury in spleen only, of whom 213 underwent CT.

**Table 1:** Patient characteristics; liver injuries, Splenic injuries, and patients with injuries to both organs (combined) with CT available, CT findings of extravasation and management.* two cases with active extravasation in both organs.

Characteristics	liver (All patients)	Spleen (All patients)	Liver and Spleen	
No. of patients (n)	329	262	49	
Age (years)	35.8 (15–85)	40.1 (15–92)	34.6 (15–66)	
Sex				
Male	212 (64.4%)	204 (77.9%)	39 (79.6%)	
Female	117 (35.6%)	58 (22.1%)	10 (20.4%)	
ISS	22 (4–75)	25 (4–75)	34 (9–75)	
ISS > 15 (n)	239 (72.6%)	216 (82.4%)	47 (95.9%)	
NISS	27 (4–75)	29 (4–75)	41 (12–75)	
NISS > 15 (n)	263 (79,9%)	232 (88.5%)	48 (98%)	
LOS days	15.8 (1-180)	13.4 (1-105)	24.4 (1-105)	
30-day survival (reciprocal of 30-day mortality)^1,2,3^	304 (92.4%)^1^	246 (93.9%)^2^	44 (89.8%)^3^	
CT findings, extravasation (n/% of total)	19 (5.8%)	47 (17.9%)	11 (22.4%)*	
Management			**Liver**	**Spleen**
Angioemblization	2 (10.5%)	24 (51.6%)	1 (20%)	3 (37.5%)
NOM	12 (63.2%)	10 (21.3%)	2 (40%)	3 (37.5%)
Packing	5 (26.3%)	-	2 (40%)	-
TachoSil	-	1 (2.1%)	-	-
Splenectomy	**-**	12 (25.5%)	-	2 (25%)

Values are given in mean (age, LOS) or median (ISS, NISS). Range in parentheses.

ISS = Injury Severity Score; NISS = New Injury Severity Score; LOS = hospital length of stay.

^1, 2, 3^ This information was missing in five, two, and one case, respectively.

Furthermore, 65 patients (65/409; 15.9%) with liver injury also had splenic injury, and 49 (49/329; 14.8%) of them underwent CT. 65 patients (65/313; 20.8%) with splenic injury also had liver injury, of whom 49 (49/262; 18.7%) underwent CT.

In Supplemental Table 1, the numbers and proportions of liver, splenic and combined injuries per year are presented.

### 30-day survival rate

The 30-day survival rate was 92%, 94% and 90% in the liver, splenic and CG- groups, respectively (Table 1). The CG had lower average age compared with the single organ injury group, higher ISS score (*p* < 0.0001) and, longer stay at the hospital (*p* < 0.001) (Table 2). However, when comparing CG with the single organ group, the 30-day survival rate was not decreased in the CG group, OR 1.32 (95% CI 0.627–4.58, *p* = 0.36).

**Table 2:** Comparison of patient characteristics between single organ liver or Splenic injuries, and injuries to both organs. CT available.

Characteristics	Liver or Spleen (Combined organ injury patients excluded)	Liver and Spleen	*p*
No. of patients (n)	493	*49*	
Age (years)	38.4 (14–92)	*34.6 (15–66)*	0.0197
Sex			0.142
Male	338 (68.6%)	*39 (79.6%)*	
Female	155 (31.4%)	*10 (20.4%)*	
ISS	21 (4–75)	*34 (9–75)*	< 0.0001
*ISS > 15 (n*)	361 (73.2%)	*47 (95.9%)*	0.001
NISS	27 (4–75)	*41 (12–75)*	< 0.0001
*NISS > 15 (n)*	399 (80,9%)	*48 (98%)*	0.0012
LOS days	12.8 (1-180)	*24.4 (1-105)*	< 0.001
30-day survival (reciprocal of 30-day mortality)^1,2^	462 (93.7%)^1^	*44 (89.8%)* ^*2*^	0.359

Values are given in mean (age, LOS) or median (ISS, NISS). Range in parentheses.

ISS, Injury Severity Score; NISS, New Injury Severity Score; LOS, hospital length of stay.

^1, 2^ This information was missing in five and in one case, respectively.

### Patients with CT performed

Of the 409 liver injury patients, 329 (80.4%) had a CT and a CT/surgically verified liver injury. The frequency of trauma CT protocols with regards to contrast phase, for cases with liver injuries, is presented in Table 3. Liver injury grading of the cohort according to AAST OIS 1994 and 2018 revisions are presented in Table 4. One patient had a liver packing before CT, so CT grading was not performed.

**Table 3:** Trauma CT protocols for liver injuries.

CT Protocol/Phase	*N*	(%)
Venous only	156	47.4
Arterial and venous	155	47.1
No contrast, arterial and venous	7	2.1
Single bolus	3	0.9
Arterial and delayed images and later a venous phase	3	0.9
Arterial and delayed images	2	0.6
Arterial only	1	0.3
Venous and arterial	1	0.3
Venous and delayed images	1	0.3
**Total**	329	100%

**Table 4:** Liver injury grading according to AAST OIS 1994 and 2018 revisions.

GRADE (1994)	*N* (%)	*N* (%)^a^	*N* (%)^b^	GRADE (2018)	*N* (%)	*N* (%)^b^
**I**	35 (10.7)	37 (14.1)	42 (6.0)	**I**	35 (10.7)	42 (6.0)
**II**	127 (38.7)	67 (25.)	91 (12.9)	**II**	117 (35.7)	91 (12.9)
**III**	128 (39.0)	107 (40.7)	215 (30.6)	**III**	135 (41.2)	210 (29.9)
**IV**	33 (10.1)	45 (17.1)	66 (9.4)	**IV**	35 (10.7)	74 (10.5)
**V**	5 (1.5)	7 (2.7)	8 (6.6)	**V**	6 (1.83)	15 (2.1)
**TOTAL**	**328** ^**c**^	**263**	**432**		**328** ^**c**^	**432**

^a^ and ^b^; data are from reference 16 and 17, respectively. ^**c**^ One patient had a liver packing before CT.

#### Prevalence of active bleeding and CVI

**Fig. 2:**
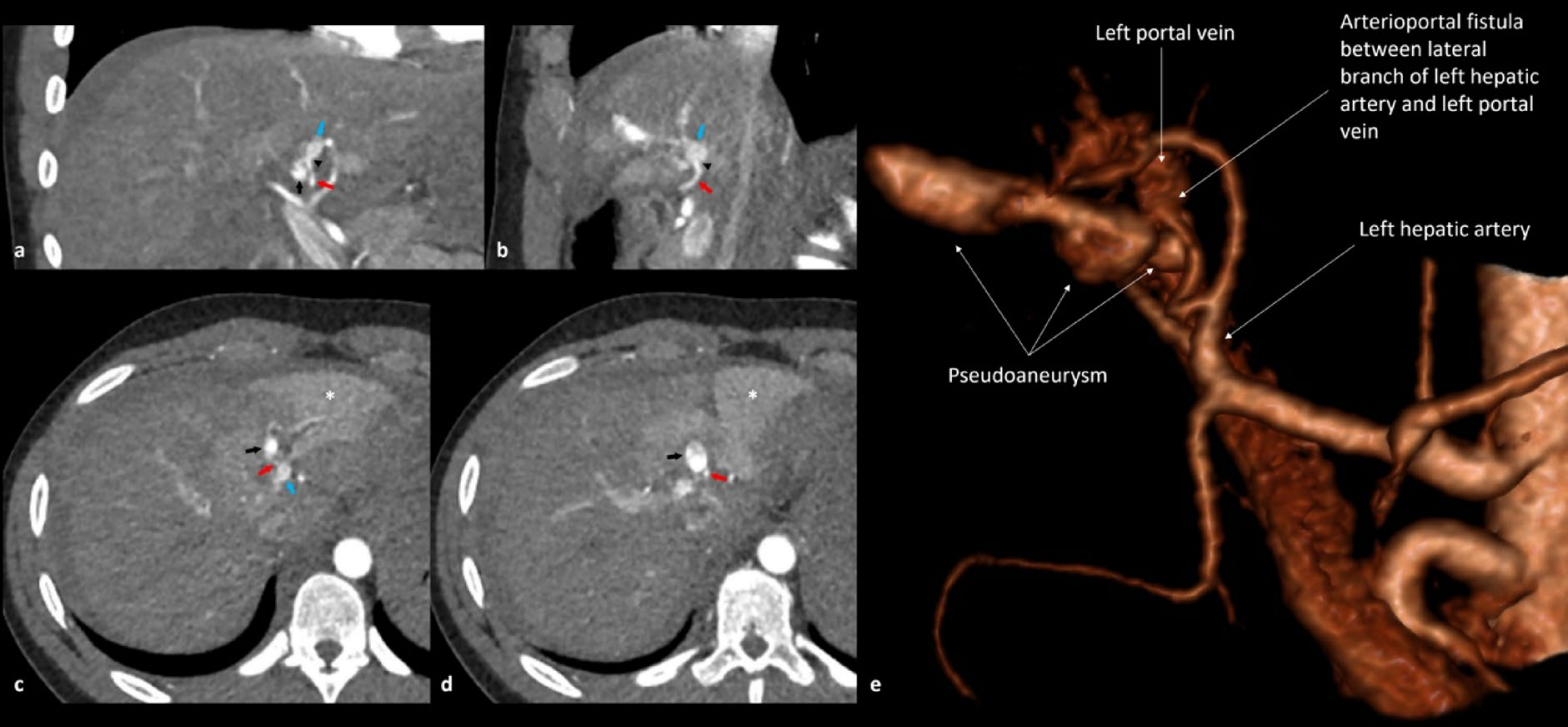
Parasternal right-sided stab wound with injury to the liver. Images **a**-**d** show the trauma CT scan in arterial phase in coronal **(a)**, sagittal **(b)**, and axial views (**c** and **d**). There is an arterioportal fistula (black arrowhead) between the lateral branch of the left hepatic artery (red arrow) and the left portal vein (blue arrow) with formation of a pseudoaneurysm (black arrow) extending along the stab wound tract in the left liver lobe. Note the arterioportal fistula associated transient hepatic attenuation difference (THAD) of the left liver lobe (white asterisk). e) The image represents the corresponding 3D rendering of the same CT scan. The arterioportal fistula (white arrow), the left portal vein (white arrow), the pseudoaneurysm (white arrows) and the left hepatic artery (white arrow and text) are shown in the image. Non-operative management was applied.

Among the subjects with liver injury, 19 patients had active bleeding (19/329; 5.8%) (Tables 5 and 6). CVI was present in four patients, of whom three had both pseudoaneruysm (PSA) and AV-fistula (Fig. 2). One patient had both active bleeding and CVI. In three cases, the trauma mechanism was motor vehicle accident (PSA only), and stabbing in three (AV-fistula and PSA).

**Table 5:** Comparison between patients with and without active bleeding in patients with liver injury who underwent CT.

CT available (*n* = 351)	Active bleeding (*n* = 19)	No active bleeding (*n* = 310)	*p*
Gender (M/F)	11/8	201/109	0.49
Age	42.1 (16–83)	35.4 (14–85)	0.09
ISS	34 (10–75)	22 (4–75)	0.025
NISS	41 (10–75)	27 (4–75)	0.045
LOS days	25.7 (2-180)	15.0 (1-140)	0.28
30-day survival (reciprocal of 30-day mortality)	18 (95%)	286 (92.3%)*	0.62

* Five patients with no information available.

**Table 6:** Comparison between patients with and without active bleeding in patinets with combined injuries with CT available.

Combined injuries CT available (*n* = 49)	Active bleeding (*n* = 11^1^)	No active bleeding (*n* = 38)	*p*
Gender (M/F)	6/5	33/5	0.033
Age	38.4 (16-57.8)	32.6 (15–65)	0.354
ISS	48 (26–75)	34 (9–75)	0.073
NISS	48 (34–75)	41 (12–75)	0.21
LOS days	22.4 (8–56)	24.5 (1-105)	0.98
30-day survival (reciprocal of 30-day mortality)	10 (90.9%)	34 (89.5%)*	1

* in one patient no information was available.

^1^ in two patients active bleeding was seen in both organs.

Based on our previous cohort on splenic injuries^14^, the prevalence of active bleeding in the spleen was 17.9%. OR for having a CVI in spleen^14^ compared with liver was 6.71 (95% CI; 2.27–19.90, *p* < 0.0001).

Of the 19 patients with active bleeding, 15 were intraparenchymal and 4 were intraperitoneal. The group with active bleeding did not differ in age but had higher ISS compared to patients without active bleeding (median (range) 34 (9–75) vs. 22 (4–75), *p* = 0.025, Table 5).

**Fig. 3:**
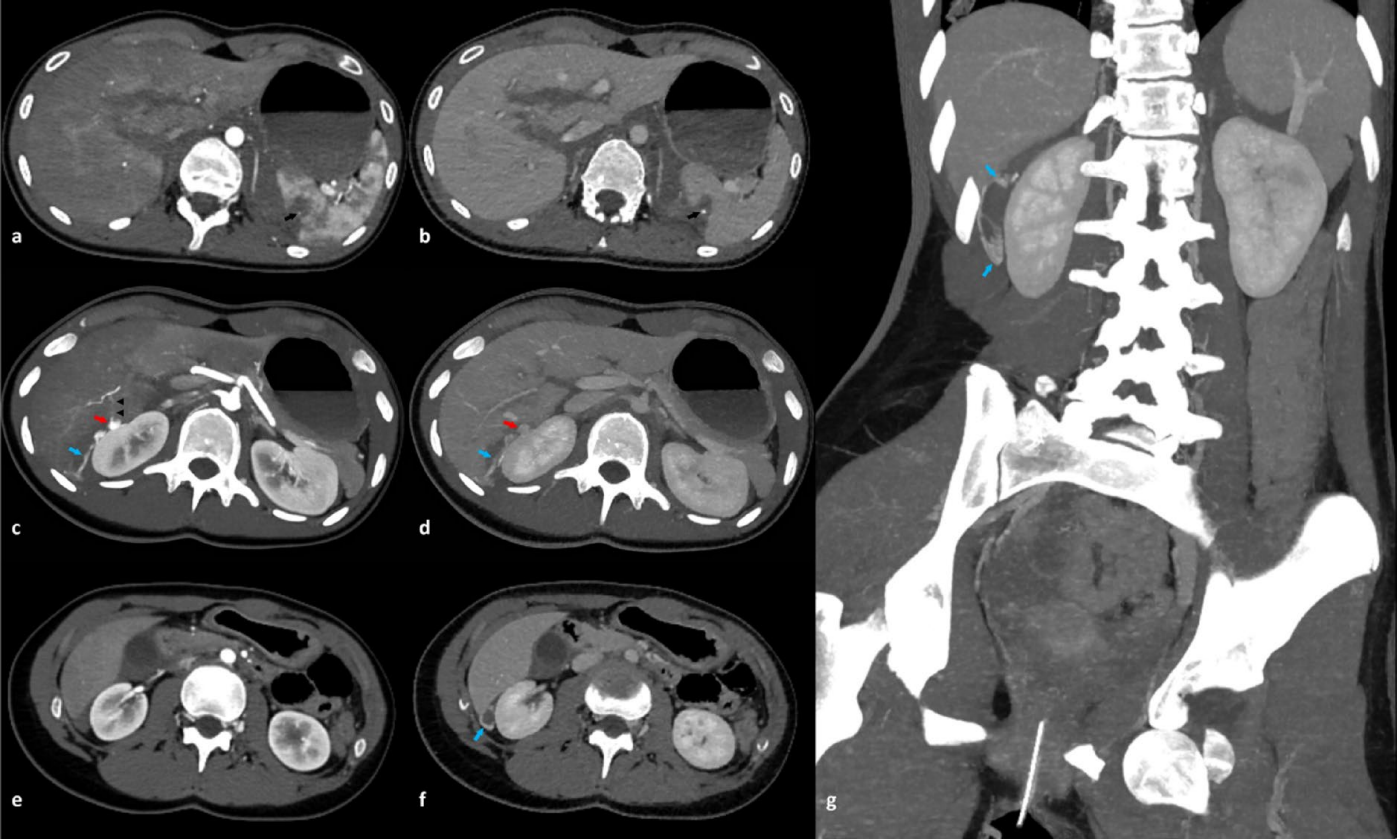
Motor vehicle collision at a speed of 180 km/h. The first column of images (**a**, **c**, **e**) is in arterial phase and the second column of images (**b**, **d**, **f**) is in venous phase. There is an active bleeding in a spleen laceration (black arrow in images **a** and **b**). There is a pseudoaneurysm (red arrow in maximum intensity projection images **c** and **d**) from a posterior branch of the right hepatic artery (black arrowheads in image c). There is an active intraperitoneal bleeding extending into Morrison’s pouch (blue arrows in images **c**, **d**, **f**, and **g**) from the pseudoaneurysm. Also, note the left hip dislocation with a Pipkin fracture. Splenic embolization was performed (not shown). For liver non-operative management was applied.

In CG, active bleeding (in liver, spleen or both) was seen in 11 (11/49 = 22.4%) subjects. In two cases, active bleeding was present in both organs (2/49; 4.1%) (Fig. 3). Five cases had active bleeding in the liver (3/5; 60% intraparenchymal) and 8 had active bleeding in the spleen (5/8; 62.5% intraparenchymal, 3/8; 37.5% intraperitoneal). This group trended towards a higher ISS score compared to the group with no active bleeding (median (range) 48 (26–75) vs. 34 (9–75) *p* = 0.073, Table 6). In CG, females had a significantly higher risk of having an active bleeding compared to males (OR 5.5; 95% CI 1.210-25, *p* = 0.033).

### Active bleeding management

The treatments for the liver or splenic injuries with active bleeding are presented in in Table 1. The most common approach for liver injuries was non-operative management (NOM) (12/19; 63.2%) (Table 1). For splenic injuries it was angioembolization (24/47; 51.1%), followed by splenectomy (12/47; 25.5%) and NOM (10/47; 21.3%) (Table 1). In two cases, where active bleeding was present in both organs, splenic angioembolization was performed in one case, while in the other case NOM was applied (Table 1).

NOM was applied to the patient with PSA only. In the three patients with both PSA and AV-fistula, one had NOM, and two had angioembolization^2^ or hemostatic agent (Floseal→)^1^ applied.

### Mechanism of trauma injury

In Supplemental Tables 2–4 the mechanisms of injury are summarized. For all liver injuries, fall from high height (high-energy) (94/329; 28.6%) was the most common cause. For the CG, motor vehicle accident (MVA) was the most common cause (15/49; 30.6%), followed by fall from high height (14/49; 28.6%).

## Discussion

The major aim of this study was to assess the prevalence of liver injuries as well as concomitant injuries to the liver and spleen seen on admission CT in patients with blunt or penetrating abdominal trauma in a single level 1 trauma center’s large, unselected material. In our nine-year series of 2805 patients, we found a total of 329 patients with liver injuries and CT available, rendering a prevalence of 11.7%. The majority of the liver injuries were classified as grade 1–3, and around 12% were grade 4 or 5. The detailed description of splenic injuries have been reported previously^13^.

49 patients had combined liver and splenic injuries, resulting in a prevalence of 1.7%, which is in line with the previously reported 1.7% among 664 patients^16^. Of our 591 liver and/or splenic injuries, 49 (8.3%) were combined and 493 (83.4%) patients had a single organ injury (i.e. injury to either spleen or liver). Two previous studies reported slightly greater proportions, with 83/80 out of their 814/703 patients with splenic or hepatic injuries (10.2%/11.4%) presenting with combined injuries^12,17^.

The CG had a lower average age compared with the single organ injury group, and significantly higher ISS score. In almost one third of patients in CG, the injury mechanism was fall from high height, followed by motor vehicle collision accident, which accounted for one fourth of the cases. Active bleeding was more common in combined injuries compared with single organ injuries. Although survival rates did not differ between those with and without active bleeding in the cohort, 30-day survival rate was slightly decreased in the entire CG compared with single organ groups.

In a recently published study from our group, the prevalence of CVI in splenic injures was 8.3%^15^, which is far greater then the prevalence of CVI in liver injuries of 1.0% (4/409, 1.2% if CT) observed in this study.

The most common treatment of active bleeding in our cohort, seen in more than 60%, was NOM; packing was performed in one fourth of the cases, and angioembolization in only 10%. Ruscelli et al. concluded that NOM for blunt hepatic (and splenic injuries) in stable or stabilizable trauma patients seems to be the choice of treatment regardless of injury grade according to the AAST Organ Injury Scale^18^. This is in line with a recent study by Nguyen PD et al., which showed that NOM in stable patients with blunt liver trauma had a similar risk of liver-related complications and mortality compared to those treated with angioembolization first^19^. These findings support that a NOM-first approach should be considered a routine strategy in blunt liver trauma when patients are stable. However, when liver trauma is severe, it is an independent predictor of severe haemorrhage with an increased risk of sepsis and trauma-related death^20^. It is crucial to distinguish between patients suitable for conservative management and those requiring interventional or surgical treatment. This requires extensive clinical experience. Notably, for high-grade liver injuries, level 2 trauma centres were less likely to use angiography compared to level 1 centres and had a higher in-hospital mortality^21^. The addition of the arterial phase to the venous phase in the CT assessment of patients with high-energy trauma, implemented at our department in 2015, likely played a significant role in increasing the detection and characterization of traumatic active bleeding^22^.

Our prevalence figures of single organ liver injuries are lower compared to another recently published study by Mukharjee et al., where the authors found liver and splenic injuries to be the most common in blunt abdominal trauma with an incidence of 60% and 52%, respectively^3^. The finding of combined splenic and liver injury was, not surprisingly, the most common combined injury with an incidence of 9.2%.

Both ISS and NISS have been shown to predict intensive care unit (ICU) admission and LOS in trauma patients^23,24^. Major trauma is commonly defined as an ISS score above 15^25,26^. Both in the liver injury group and CG, relatively high ISS scores (median 25 and 41, respectively) were found. Our findings do not differ substantially from equivalent trauma cohorts. In a similar abdominal trauma material from Norway, the overall ISS was 21 and overall 30-day survival 89%^2^. Liver injuries were the most frequent injuries, found in 38% of injured patients^2^. Afifi et al. also found 38% liver injuries in their blunt abdominal trauma material, of whom 23% underwent emergent surgery in terms of packing, resection debridement, left lobe hepatectomy and splenectomy^27^. Their overall mortality was 7.8%.

Combined injuries (liver and spleen) have been shown to have longer ICU stay and LOS, more transfusion requirements and increased mortality compared with single organ injury^13^. This is in line with our findings where CG had longer LOS (*p* < 0.001) and a slightly lower 30-day survival rate (89.8 vs. 93.7%; (*p* = 0.359) compared with single organ injury group. Patients presenting with concomitant injuries with a high ISS score, apart from having a higher incidence of active bleeding, might also have thoracic and/or head injuries that could affect the outcome^28^. In patients with damage control surgery after abdominal trauma, both higher ISS score and resuscitative thoracotomy were independent predictors and associated with increased mortality^29^.

The primary limitation of this study was, apart from the retrospective study design, that all subjects did not undergo CT. Furthermore, different radiologists performed the initial evaluation of the CT, and some patients might have been lost to follow-up. Also, the indications for whole-body trauma CT may vary considerably across institutions due to differences in thresholds for vital parameters, trauma mechanisms etc^30^. This variability may limit the direct reproducibility of our findings across trauma centers.

In conclusion, the prevalence of liver injuries with CT in our material was 11.7%. Concomitant splenic and liver injury was seen in 1.7% of blunt or penetrating abdominal trauma cases, with the majority being men. This gender distribution reflects the overall male-to-female distribution in our material. The ISS was doubled in concomitant injury group compared with single organ injury patients. We propose that a fall from high height and a high ISS shall prompt the exclusion of combined liver and splenic injuries in abdominal trauma patients. Active bleeding was seen in one fourth of patients with concomitant injury, but this did not increase the odds of not surviving compared with those without active bleeding.

## Supplementary Information

Below is the link to the electronic supplementary material.

Supplementary Material 1

## Data Availability

The datasets generated and/or analysed during the current study are not publicly available due to sensitive nature of the research and IRB restrictions but are available from the corresponding author on reasonable request.

## Abbreviations

AAST: American Association for the Surgery of Trauma
AV-fistula: Arteriovenous fistula
CG: Combined group (of liver and splenic injuries)
CT: Computed tomography
CVI: Contained vascular injury
ISS: Injury severity score
LOS: Length of hospital stay
MVA: Motor vehicle accident
NISS: New injury severity score
NOM: Non-operative management
OR: Odds ratio
RIS: Radiological information system
PACS: Picture archiving and communication system
PSA: Pseudoaneurysm
