# Supplementary material for: Prevalence, characteristics and treatment of concomitant injury to liver and spleen with vascular injury after blunt abdominal trauma
Source: Sci Rep. 2025 Aug 7;15:28972. doi: 10.1038/s41598-025-14113-w (PMC12331954; doi:10.1038/s41598-025-14113-w)
Supplement: Supplementary file 1 — Supplementary Material 1 [file 41598_2025_14113_MOESM1_ESM.docx]

| **Year** | **Abdominal injuries** | **Liver injuries^1^** | **%** | **Splenic injuries^1^** | **%** | **Combined^1^** | **%** |
| --- | --- | --- | --- | --- | --- | --- | --- |
| **2013** | 275 | 46 | 16.7 | 32 | 11.6 | 10 | 3.6 |
| **2014** | 280 | 47 | 16.7 | 31 | 11.1 | 9 | 3.2 |
| **2015** | 252 | 44 | 17.5 | 19 | 7.5 | 2 | 0.7 |
| **2016** | 268 | 35 | 13.1 | 26 | 9.7 | 3 | 1.1 |
| **2017** | 341 | 44 | 12.9 | 24 | 7.4 | 6 | 1.8 |
| **2018** | 327 | 44 | 13.5 | 14 | 4.3 | 2 | 0.6 |
| **2019** | 349 | 35 | 10.0 | 44 | 12.6 | 4 | 1.1 |
| **2020** | 368 | 46 | 12.5 | 40 | 10.9 | 7 | 1.9 |
| **2021** | 345 | 46 | 13.3 | 32 | 9.3 | 6 | 1.7 |
| **TOTAL** | **2805** | **329** | **11.7%** | **262** | **9.3%** | **49** | **1.7%** |

**Supplemental Table 1.** Number of total abdominal injuries in trauma register. Liver injuries, splenic injuries, and combined injuries, including proportions per year.

**^1^**CT available and a CT-or surgically verified liver or spleen injury.

| **MECHANISM OF INJURY** | **N** | **%** |
| --- | --- | --- |
| Fall from high height | 94 | 28.6 |
| Motor vehicle accident (MVA), not motorcycle | 52 | 15.8 |
| Penetrating injury (stab wound) | 49 | 14.9 |
| Motorcycle accident (MCA) | 37 | 11.2 |
| Blunt trauma (tree, branch, pillar, stone, other human, metal) | 33 | 10.0 |
| Bicycle accident Pedestrian accident | 23 | 7.0 |
| Pedestrian accident | 17 | 5.2 |
| Gunshot wound | 9 | 2.7 |
| Fall in the same plane (low-energy fall) | 7 | 2.1 |
| Trauma with other type of vehicle (such as electric bike) | 4 | 1.2 |
| Other | 3 | 0.9 |
| Unknown | 1 | 0.30 |
|  | **329** | **100%** |

**Supplemental Table 2. MECHANISM of INJURY.** Liver injuries for patients with CT available (n=329).

| **MECHANISM OF INJURY** | **N** | **%** |
| --- | --- | --- |
| Motor vehicle accident (MVA), not motorcycle | 15 | 30.6 |
| Fall from height | 14 | 28.6 |
| Motorcycle accident (MCA) | 7 | 14.3 |
| Pedestrian accident | 5 | 10.2 |
| Blunt trauma (tree, branch, pillar, stone, other human, metal) | 5 | 10.2 |
| Penetrating injury (stab wound) | 2 | 4.1 |
| Trauma with other type of vehicle (such as electric bike) | 1 | 2.0 |
|  | **49** | **100%** |

**Supplemental Table 3**. Mechanisms of injury. Liver and spleen both injured with CT available (n=49).

| **MECHANISM OF INJURY** | **N** | **%** |
| --- | --- | --- |
| Fall from high height | 129 | 26.2 |
| Motor vehicle accident (MVA), not motorbike | 78 | 15.8 |
| Motorcycle accident (MCA) | 67 | 13.6 |
| Penetrating injury (stab wound) | 62 | 12.6 |
| Blunt trauma (tree, branch, pillar, stone, other human, metal) | 47 | 9.5 |
| Bicycle accident | 39 | 7.9 |
| Pedestrian accident | 22 | 4.5 |
| Fall in the same plane (low-enery fall) | 16 | 3.2 |
| Gunshot wound | 15 | 3.0 |
| Trauma with other type of vehicle (such as electric bike) | 12 | 2.4 |
| Other | 5 | 1.0 |
| Unknown | 1 | 0.2 |
|  | **493** | **100%** |

**Supplemental Table 4.** Mechanisms of injury for liver or splenic injuries (single organ injury with CT available (n=493).
